# A Review on Platelet Activating Factor Inhibitors: Could a New Class of Potent Metal-Based Anti-Inflammatory Drugs Induce Anticancer Properties?

**DOI:** 10.1155/2017/6947034

**Published:** 2017-03-28

**Authors:** Vasiliki D. Papakonstantinou, Nefeli Lagopati, Effie C. Tsilibary, Constantinos A. Demopoulos, Athanassios I. Philippopoulos

**Affiliations:** ^1^Laboratory of Biochemistry, Faculty of Chemistry, National and Kapodistrian University of Athens, 15771 Athens, Greece; ^2^Institute of Biosciences & Applications, NCSR Demokritos, 15310 Agia Paraskevi, Greece; ^3^Laboratory of Inorganic Chemistry, Department of Chemistry, National and Kapodistrian University of Athens, Panepistimiopolis Zografou, 15771 Athens, Greece

## Abstract

In this minireview, we refer to recent results as far as the Platelet Activating Factor (PAF) inhibitors are concerned. At first, results of organic compounds (natural and synthetic ones and specific and nonspecific) as inhibitors of PAF are reported. Emphasis is given on recent results about a new class of the so-called metal-based inhibitors of PAF. A small library of 30 metal complexes has been thus created; their anti-inflammatory activity has been further evaluated owing to their inhibitory effect against PAF in washed rabbit platelets (WRPs). In addition, emphasis has also been placed on the identification of preliminary* structure-activity* relationships for the different classes of metal-based inhibitors.

## 1. Introduction

This work was motivated by the fact that a comprehensive survey on metal-based potent inhibitors of PAF as active anti-inflammatory drugs has not been previously described in the literature. It is the aim of this microreview to reveal the critical role of the metal center and of the molecular structure (different coordination geometries) of the relevant metal complexes within this series of new* metal-based* potent inhibitors of PAF. Biological results of these compounds are reviewed and added to the dataset base of inorganic metal-based anti-inflammatory drugs. The review is divided into two general parts. At first, the general characteristics of PAF are described, followed by selected, known organic inhibitors of PAF. In the second part, the structural characteristics and the biological activity against PAF, of different classes of metal-based inhibitors, are presented.

## 2. General Characteristics of the Platelet Activating Factor

### 2.1. Platelet Activating Factor, Structure, and Activity

**Figure 1:**
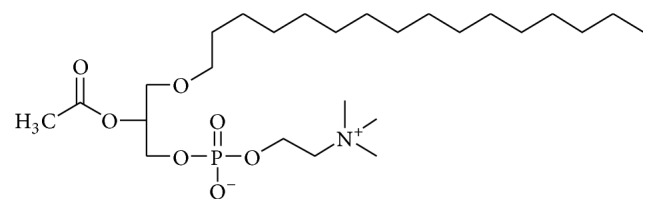
PAF molecular structure.

Platelet Activating Factor (PAF) has been characterized as a new, ubiquitous, potent, and unique class of lipid chemical mediators that share similar biological activities, namely, PAF-like activity molecules [1]. Originally, the term PAF was meant to be one phosphoglycerylether lipid, identified as 1-O-alkyl-2-acetyl-*sn*-glycero-3-phosphocholine with 16–18 carbons at the ether-bonded fatty chain at the sn-1 position of the glycerol backbone, as shown in Figure 1 [2]. Interestingly, while most of the ether lipids replaced their ether bond with esterified analogues through time, PAF conserved its ether bond in sn-1 position because of its important biological role [3].

PAF is produced by a plethora of cells as platelets, neutrophils, monocytes/macrophages, lymphocytes, basophiles, eosinophils, mast cells, and endothelial cells and has many biological roles, both physiological and pathological depending mainly on the extent of PAF production and enzymatic regulation [4]. Under physiological conditions, the production of PAF is regulated by its enzymes [5] and the produced PAF participates in physiological processes as reproduction, memory formation, vascular tone, apoptosis, and angiogenesis. However, in pathological conditions, excess amounts of PAF can cause inflammation and lead to inflammatory conditions or diseases, such as allergy, asthma, atherosclerosis, diabetes, renal diseases, cancer, HIV pathogenesis, and periodontitis [6].

### 2.2. PAF Receptor

PAF exerts its autocrine and paracrine actions through binding to a well-characterized G-protein coupled receptor (GPCR) located on the plasma membrane of a wide variety of mammalian cells such as endothelial cells, neutrophils, monocytes, dendritic cells, platelets, and leukocytes [7]. There are few studies that prove the existence of PAFR in endomembranes as well. The first one revealed three distinct classes of binding sites for PAF. Two of them were found on the microsomes and the third one was on the synaptic plasma membranes of rat cerebral cortex [8]. Later, other studies certified the intracellular existence of PAFR as well as the presence of PAF binding sites in the nucleus, both at the nuclear envelope and at nuclear matrix [9–11].

Finally, it seems that cytoplasmic PAFR is implicated in the acute effects of PAF, while the intracellular PAFR intermediates the long-standing effects of PAF via the modulation of gene expression.

The interaction of PAF with its receptor (PAFR) initiates a cascade of diverse intracellular signaling pathways, which translate the mediator's message to the final cell response [12]. The signal transduction pathways activated by PAF are cell and species dependent and are regulated by several mechanisms. These include covalent modification and the internalization of PAFR, the modulation of PAFR gene expression, and binding potential along with the fine-tuning of the signal transduction pathways by a plethora of intracellular and extracellular modulators [6].

### 2.3. PAF Inhibitors

There are hundreds of molecules that are able to inhibit biological actions, mediated by PAF. Most of them have been screened in vitro in cell systems and animal models and the ones with the best properties, in terms of potency, bioavailability, and safety, have been also tested in clinical trials. There are many ways to classify PAF inhibitors such as (i) their source, isolated from natural sources or chemically synthesized; (ii) their chemical structure, nitrogen heterocyclic compounds, PAF analogues (PAF-like molecules), dihydropyridines, natural medicines, and others; or (iii) the way they interact with PAF receptor, specific or nonspecific inhibitors. IC_50_, of various PAF inhibitors against PAF aggregation in washed rabbit platelets, are presented in Table 1. In the present minireview, PAF inhibitors will be divided into a new classification method as organic and inorganic.

## 3. Organic PAF Inhibitors

### 3.1. Phytochemical Products

**Figure 2:**
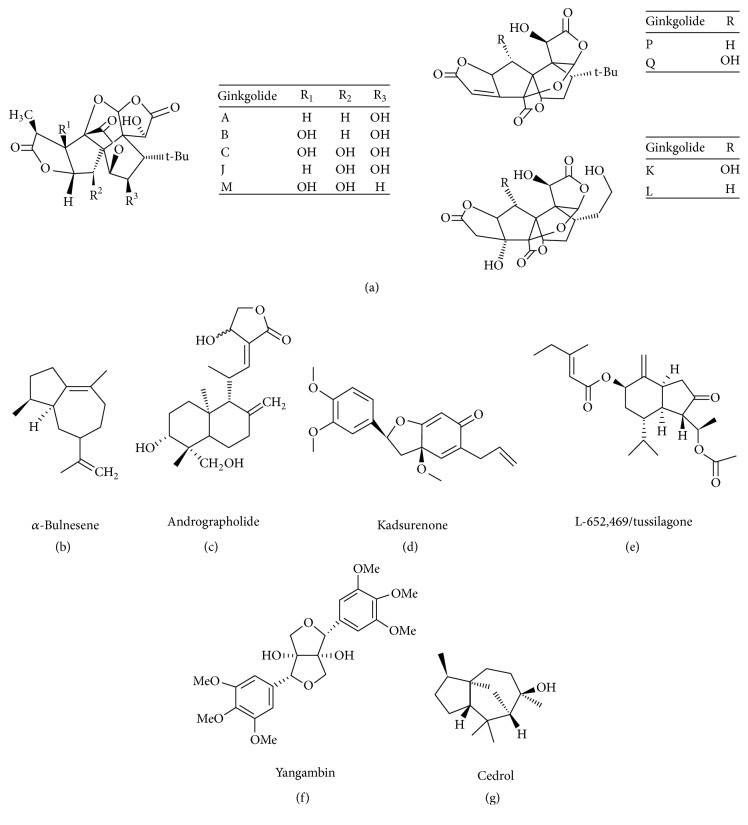
Phytochemical products with anti-PAF activity.

There are several hundreds of phytochemical products that can inhibit PAF biological activities and therefore act as anti-inflammatory agents. The purpose of this review is not to list all of them, so below are some of the most promising ones for therapeutic use or dietary supplements (Figure 2). Ginkgolides are diterpenes with a structure of twenty carbon cage molecules that come from the leaves and the roots of an ancient Chinese tree called* Ginkgo biloba* and belong to the natural specific inhibitors which antagonize the binding to PAF's membrane receptor by a competitive way. Based on the molecular dynamics simulations, the binding of PAF to PAFR leads to its activated state, while the binding of* Ginkgo biloba* locks PAFR in its inactive state. There is a plethora of pathological conditions, where PAF is implicated, and* Ginkgo biloba* extracts have managed to ameliorate, as cognitive disorders, HIV infection, ischemia, tissue injuries, cancer, and airway diseases as asthma and allergy [13].

Andrographolide has a labdane diterpenoid structure and it is obtained from the stems and the leaves of a Asian plant called* Andrographis paniculata *which was traditionally used against viral infections and inflammatory diseases [14]. The studies around its inhibition against PAF have revealed that it is a nonspecific PAF inhibitor [15] with a plethora of promising effects as anti-inflammatory, anticancer, immunomodulatory, antiviral, and cardioprotective [16].

Alpha-bulnesene is a sesquiterpenoid molecule that comes from the essential oil of an Asian plant called* Pogostemon cablin* and acts as a PAF inhibitor exerting its inhibitory effect by antagonizing competitively PAF binding to its receptor and thus inhibits intracellular Ca^2+^ increase [17]. As it is a potent anti-inflammatory agent, it may be used for the inhibition or prevention of allergic syndromes, providing new antiallergic medicine that exhibits fewer side effects [18].

*Kadsurenone* is the first potent PAF antagonist that was discovered and comes from the Chinese herb* Piper futokadsurae*. It is a benzofuranoid neolignan whose structure was used as a template for the development of synthetic PAF antagonists as well as for the molecular modeling of PAFR [19].

Tussilagone (or L-652,469) is a terpene that comes from the buds of the plant* Tussilago farfara *and acts as a nonspecific PAF inhibitor by blocking the calcium channels [20]. It is extensively used in inflammatory respiratory diseases including cough, asthma, and acute/chronic bronchitis because of its anti-inflammatory properties [21].

Yangambin is a natural furofuran lignan, isolated from the Brazilian plant* Ocotea duckei Vattimo. *Studies have revealed that it is a specific PAF inhibitor which antagonizes competitively PAF binding to its receptor [22]. It displays beneficial actions against allergy and headache [23, 24] along with its cardioprotective role [25].

Lipid extracts from the plant* Urtica dioica*, the common nettle, were found to contain both PAF and PAF-like activity molecules and glycolipid derivatives which act as PAF inhibitors and hence explain the sense of urticaria and the beneficial effects of nettle as traditional allergy relief and diuretic remedy [26].

The lipid molecule, di-hydroxy-chimyl-alcohol, derived from pine pollen, was also found to exert anti-PAF action [27].

Cedrol is a sesquiterpene alcohol which is found as an essential oil in many plants as* Biota orientalis*,* Pterocarpus indicusirginia*, in various types of conifers as* Cupressus* and* Juniperus*, and in* Origanum onites* [28]. It is one of the well-known PAF antagonists [29].

### 3.2. Synthetic Products

**Figure 3:**
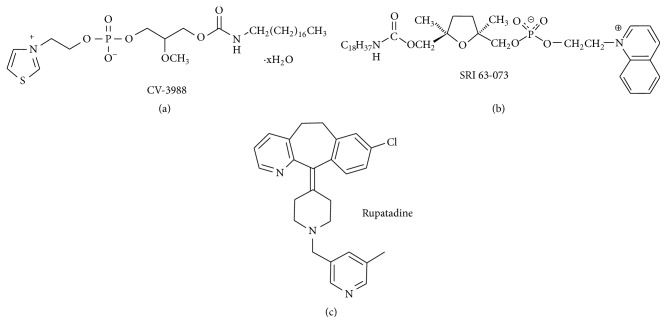
Synthetic PAF inhibitors.

The promising results of natural PAF inhibitors evoke the need for the development of synthetic PAF inhibitors. The first molecules that were synthesized had similar chemical structure with PAF, meaning a glycerol backbone such as CV-3988 [30], CV-6209 [31], ONO-6240 [32], and Ro 19-3704 [33]. The idea that followed was to replace the glycerol backbone with a cyclic structure such as SRI 63-073 [34], SRI 63-441 [35], UR-11353 [36], and CL-184,005 [37] (Figure 3).

Later, the PAF inhibitors that were synthesized had no similar structure to PAF. These molecules contain heterocyclic structures which are characterized by sp^2^ nitrogen atom that is able to interact with PAFR as a hydrogen bond acceptor. These kinds of inhibitors are pyrrolothiazole-related antagonists as tulopafant [38], thiazolidine derivatives as SM-10661 [39], imidazolyl derivatives as modipafant [40], and lexipafant [41] and hetrazepine derivatives as WEB-2086 and WEB-2170 [42]. All the above synthetic antagonists display a great variability in their chemical structure which might have importance in their different pharmacological profile.

The success of the in vitro studies of PAF inhibitors in combination with the use of many natural PAF inhibitors as traditional remedies for inflammatory diseases had been very promising for the use of PAF inhibitors in the clinical practice. Many PAF inhibitors were tested in clinical trials displaying tolerability and safety but with no effectiveness. Modipafant [40], UK-74,505 [43], WEB 2086 [44], and SR27417A [45, 46] showed no effect against asthma disease. Lexipafant, one of the most potent PAF inhibitors, was tested against cognitive impairment [47], myocardial infraction [48], sepsis [49], and organ failure in severe pancreatitis [50] with no significant results. Moreover, SR27417A did not decrease the symptoms of acute ulcerative colitis [51]; BN 50730 showed no significant amelioration of rheumatoid arthritis [52] as well as Ro 24-238 against psoriasis [53] and TCV-309 against septic shock [54]. However, there are also trials with positive results such as UK,74505 on airway and systemic responses [55], levocetirizine in chronic idiopathic urticaria [56], WEB 2086 gel against UVB-induced dermatitis [57], BN 52021 on pulmonary function in the early postischemic period [58], and Y-24180 in bronchial hyperresponsiveness in patients with asthma [59]. Last but not least comes the use of rupatadine against allergic rhinitis and several other allergic disorders [60], the positive outcomes of which resulted in the production of the first circulating drug based on a PAF inhibitor, named Rupafin.

In addition, molecules demonstrating dual antagonistic actions against PAF as well as against another inflammatory mediator have been synthesized and their therapeutic properties have been studied in vitro. These molecules inhibit both PAF actions and 5-lipoxygenase, as LDP-392 [61], or thromboxane synthase [62], or iNOS induction [63]. Rupatadine, as well, is both an oral PAFR antagonist and a histamine H(1)-receptor antagonist [64].

As far as it concerns already used pharmaceutical products, there are many of them that were found to exhibit potent inhibitory effect against PAF actions, which is expected as Platelet Activating Factor is implicated in inflammatory processes and therefore in a plethora of pathological conditions with inflammatory background. PAF is known to be implicated in atherosclerosis [65] and many statins as well as digoxin, which are used for their cardioprotective properties, are also PAF inhibitors [66, 67]. Many of the antiretrovirals used for HIV infection are also PAF inhibitors, both in vitro and in vivo [68–72].

### 3.3. Diet Products

The regulation of PAF actions is also succeeded by the daily ingestion of PAF inhibitors which are consumed through our diet (see Figures S1 and S2, in the Supplementary Material available online at https://doi.org/10.1155/2017/6947034). Among all diets, the Mediterranean diet consists of numerous products that exert potent anti-PAF activities in the washed platelet aggregation assay, such as virgin or regular olive oil [73, 74], wine and grapes [75–78], honey [79], fish [80–82], milk and yoghurt [83], and traditional Greek meals [84], the isolation and structural characterization of which demonstrated the presence of complex polar lipids as glycolipids and ganglioside derivatives, phenolics, and phenol glucosides [65]. Furthermore, from the acetylation of poplar lipids and phenolic compounds derived acetylated products with anti-PAF action [85, 86], especially, olive oil was found to contain high levels of PAF antagonists among other vegetable oils and the structure of its most active fraction was found to be a glycerolglycolipid [74]. In another study, olive pomace, crude olive pomace oil, and waste byproducts from olive pomace oil production were found to contain polar lipids as bioactive compounds that inhibit or antagonize PAF. The most bioactive compound came from olive pomace and has been chemically characterized as a glycerylether-sn-2-acetyl glycolipid [87]. Moreover, microconstituents of several seed oils, such as sesame, corn, and sunflower, were found to have either inhibitors or agonists of PAF. The most active agonist came from corn oil and was found to be almost five orders of magnitude less active than PAF, meaning that its action, in several cells and/or tissues, through PAF receptors, would minimize the biological effects of PAF [88]. In vivo, polar extracts from olive oil and olive pomace have reduced the atheromatic lesion in hypercholesterolemic rabbits [89, 90] and mellitus patients reduced platelet sensitivity after one month of traditional Mediterranean diet [84], while wine consumption improved platelet sensitivity independently of alcohol [91]. Considering the expanding role of PAF and other lipid mediators in human pathophysiology, the study of their dietary modulation is of increasing scientific interest. Today, a plethora of studies report molecules that inhibit the cellular effects of PAF in vitro and in experimental animal models or human studies in vivo, which are well presented in the review of Nomikos et al. [92].

Apart from Mediterranean products, PAF inhibitors can also be found in many other products as garlic [93–95], soy sauce [96], and tea [97]. Last but not least comes curcumin which exerts potent antiplatelet activity through the inhibition of COX and the blockade of calcium signaling. Additionally, curcumin managed to reduce colonic mucosal and tumor PLA_2_ [98, 99].

Referring to vitamins, paracitol (Vitamin D) is known to improve the inflammatory status of hemodialysis patients, an ability possibly due to its anti-PAF activities [100] and also tocopherol (Vitamin E) inhibits PAF-induced aggregation [101, 102]. Besides, there are endogenous PAF inhibitors as blood cardiolipin which was shown to control PAF actions [103].

The involvement of PAF in cancer is already known and especially in carcinogenesis, melanoma, or metastasis [104–107]. Also, various compounds with anti-PAF activities, as* Ginkgo biloba* extracts [108, 109], andrographolide [110, 111], tussilagone [112], WEB 2170 [113], and WEB 2086 [107], have been shown to exert anticancer properties in vitro. In addition, both cisplatin and WEB 2086 administered to animal models decreased dramatically tumor growth [114]. This manuscript studies whether a new class of potent metal-based anti-inflammatory drugs could induce indirect anticancer properties.

The hypothesis claims that the tumor cells express PAF receptor and therefore there are high PAF levels all around the microenvironment of the tumor. The use of anti-PAF compounds decreases PAF levels ergo inflammation and therefore enhances the anticancer properties of the chemotherapy. Moreover, the use of PAF inhibitors, such as metal compounds, which may have additional direct anticancer properties, will amplify the effectiveness of the common anticancer treatments.

## 4. Inorganic Metal-Based Inhibitors

As mentioned in the previous part, organic compounds represent well-known antagonists in the area, while application of metal-based compounds as PAF inhibitors has been systematically ignored, although the advantages of metal-based therapeutics over the organic analogues have been well addressed recently [115]. In fact, transition metal coordination compounds display a number of different coordination numbers and geometries (square planar, tetrahedral, and octahedral) as opposed to the typical tetrahedral geometry for the organic congeners. Moreover, metal complexes present a number of special characteristics such as wide structural diversity, the possibility of tuning thermodynamic and kinetic ligand substitution, and different oxidation states [116–118]. In addition, a number of metal ions, playing a vital role for life, are involved in many natural biological processes [119–122]. As a result, our current research interest has been focused on the synthesis of transition metal coordination compounds and their applications mainly as potent inhibitors of PAF, which may possess similar or significantly higher activity and/or selectivity in regard to the common organic-based inhibitors. This is a vast research area and work on this field has not been referred to previously. A recent review by Leung et al. [123] has highlighted some examples of transition metal complexes that have been investigated for their anti-inflammatory activity. However, a significant number of coordination compounds with potential anti-inflammatory action have not been included. In this respect, it is the aim of the present minireview to present existing* metal-based* coordination compounds that exert their inflammatory activity by blocking the expression of PAF. Preliminary* structure-activity *relationships have been established, studying diverse parameters such as (i) the coordination geometry of the metal complex (square planar versus octahedral, etc.), (ii) the nature of the bidentate ligands and occasionally of the moieties in its periphery, (iii) the effect of the counteranions, and (iv) the total charge and size of the complex.

Below, we describe the existing different categories of metal-based inhibitors of PAF which have been systematically studied. Other classes of anti-inflammatory compounds do not constitute the aim of this minireview.

### 4.1. Rhodium Complexes with Bidentate Nitrogen Containing Ligands

#### 4.1.1. Rhodium(III) Compounds

**Figure 4:**
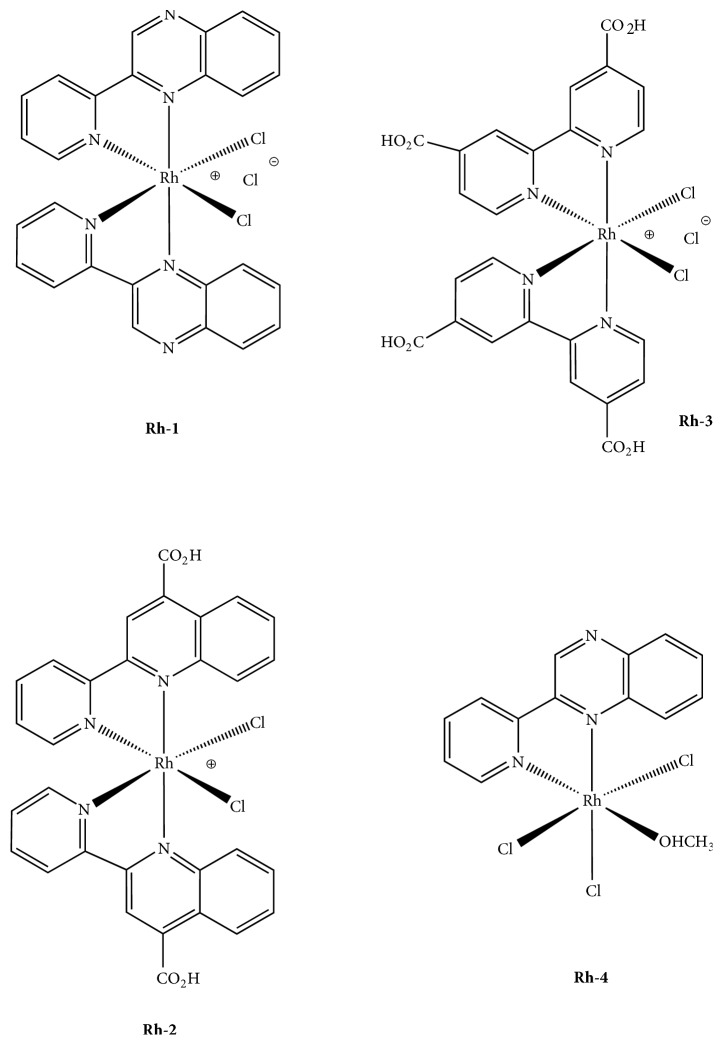
Molecular structures of rhodium(III) inhibitors of PAF.

In 2009, we have successfully reported a preliminary work [124] on the anti-PAF activity of a Rh(III) coordination compound, namely, cis-[Rh(L)_2_Cl_2_]Cl (**Rh-1**), where L stands for the bidentate ligand 2,2′-pyridylquinoxaline with an IC_50_ value of 250 nM (Figure 4). The corresponding bidentate ligand has been chosen owing to the remarkable biological properties reported [125, 126], while, on the other hand, investigation of the biological activity of inert metal complexes, such as rhodium(III) compounds, shows gradually increasing interest [127–130]. These reasons led us to design the target molecule** Rh-1** (Figure 4) with octahedral coordination geometry and evaluate in vitro its inhibitory effect against PAF-induced platelet aggregation in washed rabbit platelets (WRPs). The inhibitory effect of** Rh-1** and of all other new compounds tested (vide infra) was expressed by their IC_50_ value in *μ*M (Table 2). The IC_50_ values reflect the inhibition strength of each compound, since a low IC_50_ value reveals stronger inhibition of the PAF-induced aggregation for a given metal complex concentration.** Rh-1** shows a strong inhibitory effect towards the PAF-induced WRPs aggregation with IC_50_ = 125 nM. This is an improved value as compared to the first measurement performed (IC_50_ = 250 nM) because of slight changes in the experimental protocol during the biochemical experiment [131]. Since PAF is participating in inflammation, these compounds are potential anti-inflammatory drugs. After that, following a systematic approach [132], we have successfully developed a series of analogous rhodium(III)-based inhibitors of PAF of the general type* cis*-[Rh(L1)_2_Cl_2_]Cl (**Rh-2**) and* cis*-[Rh(L2)_2_Cl_2_]Cl (**Rh-3**) incorporating the bidentate ligands L1 = 4-carboxy-2-(2′-pyridyl)quinoline and L2 = 2,2′-bipyridine-4,4′-dicarboxylic acid that bear one and two carboxylic acid groups (-COOH), respectively, in the ligand sphere (Figure 4).

The idea, behind this, was to examine the possible effect of the carboxylic acid groups on the inhibition of the PAF, since, recently, an apparent dependence of biological activities (cytotoxicity and antioxidant efficiency) on incorporation of -COOH groups in the bipyridine ring has been reported [133]. The importance of the carboxylate moiety as a pharmacophore to the medicinal chemistry has been reported [134], while, interestingly, it has been proposed that approximately 25% of all drugs contain a COOH moiety.

The results of the biological assay performed reveal that indeed both substances (**Rh-2 **and** Rh-3**), containing different number of carboxylic acid groups in the ligand periphery, display submicromolar activity (IC_50_ = 0.35 *μ*M and 0.51 *μ*M, resp.) that is in the same order of magnitude as that of the** Rh-1** inhibitor. Evaluation of the anticancer activity of these substances is currently underway so as to check the relationship between PAF and cancer (vide infra). On the other hand, the implication of PAF in carcinogenesis and cancer metastasis has been well documented, whereas the known BN 5202 inhibitor has showed antitumor effects [135]. Cytotoxicity tests of the** Rh-1** compound against HEK-293 and cancer cell lines MCF-7 (breast cancer) were performed using cisplatin as a reference. Interestingly, preliminary results [131] reveal that** Rh-1**, a potent anti-PAF inhibitor (IC_50_ = 0.12 *μ*M), is less cytotoxic (54% viability on HEK-293 cells) compared to the cisplatin analogue (10.3% viability) with an IC_50_ value of 0.55 *μ*M against PAF. On the other hand,** Rh-1 **was proved to be less potent (77% viability) on the MCF-7 cancer cells. For cisplatin, a 16% viability was observed in this cell line, evidence of the lack of harsh toxicity of the** Rh-1** accompanied by anticancer activity. Perhaps these compounds of the chemical formulas* cis*-[Rh(L)_2_Cl_2_]Cl, that inhibit PAF action, may also be used as a potential anticancer agent.

**Figure 5:**
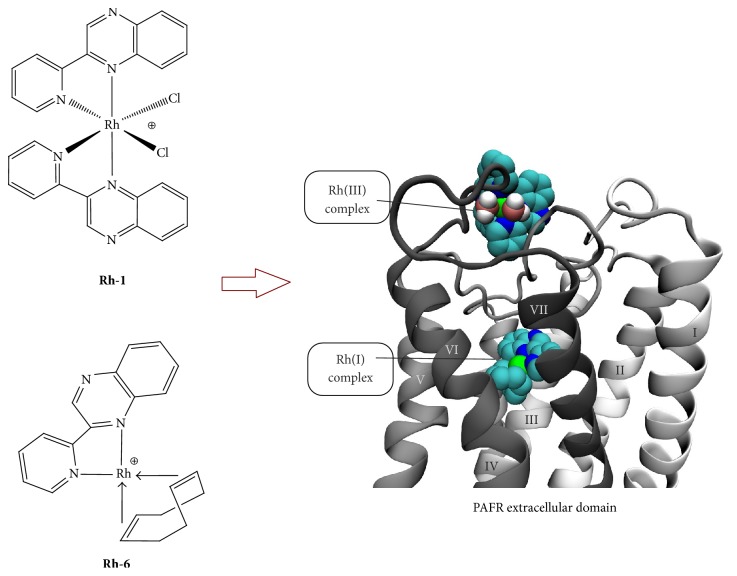
Molecular model of PAFR with the two predicted binding sites of the octahedral Rh(III) complex,** Rh-1**, and the square planar Rh(I) complexes,** Rh-5** and** Rh-6** (cartoon representation).

In an effort to get an insight about the possible mechanism of action of these substances related to the PAFR, we performed radioactivity experiments (Scatchard analysis) involving the specific binding of [^3^H]-PAF to washed rabbit platelets and its inhibition by complex** Rh-1**, that is the most potent of these Rh(III) derivatives. The specific PAF antagonist, BN 52021, was used as a reference compound and the radioactivity was measured by scintillation counting. This compound induced 18% inhibition of PAF binding onto platelet PAFR, whereas the highest %  *I* (%  *I* = [(total binding − total binding with tested compound)/specific binding] × 100) was observed at 50 nM (34% inhibition). These data suggest that** Rh**-**1** inhibits PAF action on platelets and this effect is attributed only in part to the inhibition of PAF binding onto PAFR (nonspecific binding). It seems that, at low concentrations,** 1** affects PAFR much more effectively and inhibits PAF action by another pathway [124]. Molecular docking theoretical calculations were in accord with the previous reported finding, denoting that the octahedral Rh(III) complex** Rh**-**1** (and** Rh**-**2** and** Rh-3**) with two aromatic ligands cannot fit into the ligand-binding site of the PAFR model. Instead, they could bind to the extracellular domain of the receptor (Figure 5) and, therefore, antagonize the substrate's entrance to PAFR.

From the reaction of L with one equivalent of RhCl_3_ × 3H_2_O, the [Rh(L)Cl_3_(MeOH)] complex has been prepared as a mixture of the* mer* and* fac* isomers. Upon purification the* mer*-[Rh(L)Cl_3_(MeOH)] isomer (**Rh-4**) crystallizes selectively from a mixture of both isomers in solution, while the* fac*-isomer was not isolated in a pure form.** Rh-4** is less effective (IC_50_ = 2.6 *μ*M) compared to the more bulky compounds** Rh-1**,** Rh-2**, and** Rh-3** (Figure 4). The calculated binding affinity of 3.20 *μ*M is in very good agreement with the in vitro results. As far as the rhodium(III) compounds are concerned, we conclude that the number of bidentate ligands and, as a result, the size of the target molecule (metal complex) affect dramatically the biological activity expressed. Thus,** Rh-4 **which comprises a single aromatic ligand is less effective compared to the sterically demanding** Rh-2** and** Rh-3** complexes with two ligand molecules. At the same time, the role of total charge on the metal complex and ion size cannot be ruled out as recently reported for metal complexes as inhibitors of the 26S proteasome in tumor cells [136]. The rhodium precursor RhCl_3_ × 3H_2_O and the organic ligands L, L1, and L2 have also been tested, exhibiting a weak inhibitory effect, ranging from 20 *μ*M to 67 *μ*M. From this comparison, it becomes evident the crucial role of the metal center in the final structure of these coordination compounds towards these inflammatory mediators.

#### 4.1.2. Rhodium(I) Compounds

**Figure 6:**
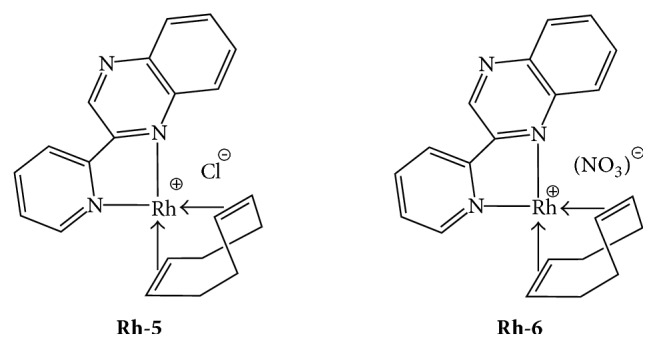
Molecular structure of Rh(I) inhibitors.

The square planar Rh(I) complexes [Rh(L)(cod)]Cl (**Rh-5**) and [Rh(L)(cod)](NO_3_) (**Rh-6**) (Figure 6) were prepared from the [Rh(cod)Cl]_2_ dimer (cod = C_8_H_12_) precursor and the corresponding ligand L [132].

The biological evaluation showed that** Rh-5** and** Rh-6** exhibit an IC_50_ value within the range of 15 to 16 nM, indicating that these organometallic compounds are* selective *and potent inhibitors of PAF in the nanomolar scale. Docking theoretical calculations support the experimental results reported, predicting a high affinity of** Rh-5 **and** Rh-6** for PAFR (estimated at *K*_*i*_ = 20 nM) that is mainly attributed to the favourable van der Waals and desolvation energy terms of its hydrophobic ligand (cyclooctadiene moiety). Finally, a clear* structure-activity* relationship is deduced, denoting that the square planar organometallic rhodium(I) complexes** Rh-5**,** Rh-6** are better inhibitors of the PAF-induced aggregation in comparison to the Rh(III) octahedral complexes** Rh-1/Rh-2/Rh-3/Rh-4**. The enhanced inhibitory activity of** Rh-5 **and** Rh-6** could be further attributed to the presence of the hydrophobic cyclooctadiene moiety (vide infra the docking experiments, Figure 6). Interestingly, the biologic activity (observed IC_50_ values) of** Rh-5** and** Rh-6** against the PAF-induced aggregation is comparable to the corresponding action of some of the most potent PAF receptor antagonists, namely, WEB 2170, BN 52021, and rupatadine (IC_50_ = 0.02, IC_50_ = 0.03 and IC_50_ = 0.26 *μ*M, resp.) [137–139]. In any case, it has to be pointed out that, upon coordination of the organic ligands (L, L1, and L2) to the rhodium center a dramatic increase* (synergetic effect)* of the inhibitory activity towards, the PAF-induced rabbit PRP aggregation is taking place in a dose-dependent manner. Therefore, the effects of rhodium metal complexes towards this inflammatory mediator can be clearly attributed to the final structure (square planar and/or octahedral) of these coordination compounds.

### 4.2. Copper(II), Zinc(II), Ni(II), and Ga(III) with Chalcogenated Imidodiphosphinato Ligands

**Figure 7:**
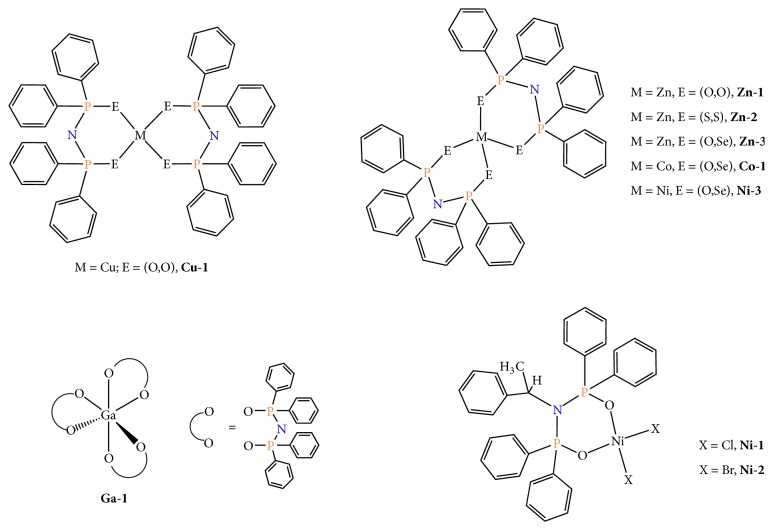
Molecular structures of metal complexes bearing PNP ligands.

Tsoupras and coworkers [140, 141] have described a series of metal complexes (M = Cu, Co, Ni, Zn, Ga) with chalcogenated imidodiphosphinato ligands showing an inhibitory effect towards PAF-induced aggregation in the nanomolar to micromolar range (vide infra). The metal complexes examined are as follows: [M{(OPPh_2_)(OPPh_2_)N}_2_], M = Cu (E = O,O;** Cu-1**); M = Zn ((E = O,O;** Zn-1**); (E = O,S); (E = S,S;** Zn-2**); (E = O,Se;** Zn-3**)); [M{(OPPh_2_)(OPPh_2_)N}_3_], M = Ga (E = O,O;** Ga-1**); [M{(Ph_2_*Ρ*)_2_N-S-CHMe}X_2_], M = Ni (E = P,P; X = Cl,** Ni-1**); (E = P,P; X = Br,** Ni-2**); (E = O,Se;** Ni-3**); M = Co, Ni (E = O,Se;** Co-1**). These complexes display diverse coordination geometries, which range from square planar for** Cu-1**,** Ni-1** and** Ni-2** to tetrahedral for** Zn-1 **and** Zn-2**, while a pseudotetrahedral environment has been found for the corresponding** Zn-3**,** Co-1**, and** Ni-3** analogues. Their molecular structures are depicted in Figure 7 and the corresponding IC_50_ values are included in Table 3. Remarkably,** Co-1** and** Ni-3** (E = O,Se) exhibit the strongest inhibitory effect against the PAF-induced aggregation in WRPs with an IC_50_ value of 18 to 19 nM. The increased biological activity of both complexes has been mainly attributed to the typical* open shell* structure they possess. It seems also that, in these ligands, the combination of O and Se, as donor atoms, is beneficial for Co(II) and Ni(II) ions over the Zn(II) ions which possess a* closed shell* structure. In fact, the biological activity of** Zn-3** drops almost two orders of magnitude (IC_50_ = 1.11 *μ*M) than that of** Co-1** and** Ni-3**. For the structurally related diamagnetic Zn(II) complexes** Zn-1** (E = O,O) and** Zn-2** (E = S,S), the presence of O or S donor atoms increases slightly the inhibitory capacity against PAF (IC_50_ = 0.54 *μ*M and 0.36 *μ*M, resp.). Moreover, the square planar** Cu-1** compound exhibited medium inhibitory effect against PAF. On the other hand, the more bulky** Ga-1** complex, consisting of a main group element and three bidentate PNP ligands with O,O as the donor atoms, shows an appreciable anti-PAF activity (IC_50_ = 62 nM). The authors have commented that the higher potency may be due to the octahedral structure of this compound. Finally, the square planar compounds** Ni-1** and** Ni-2** are the less active, owing to a partial degradation in DMSO. At this point, it should be stated that for biological purposes chemical stability in solution is a prerequisite. However, a large number of biological activity studies have been carried out so as to systematically ignore the possible effects of the corresponding medium that is used to dissolve a new substance under investigation. In particular, the presence of easily dissociating groups in a metal complex (halides, etc.) and the use of organic solvents with strong donor abilities (DMSO, DMF, and MeCN) constitute it susceptible to a possible decomposition in solution. The* nature of the active species* in solution remains unclear affecting the results of the biological experiment. In summary, the authors of this study have concluded that the stereochemical and electronic characteristics of the metal complexes previously described determine their inhibitory effect. Among the inhibitors studied,** Co-1**,** Ni-3**, and** Ga-1** could be potentially examined for their anti-inflammatory activity. It may be that these compounds express their biological activity via a selective interaction with the PAF receptor, although this has not been examined. Docking theoretical calculations might be helpful towards this aim as previously shown for the rhodium(I)/(III) series [124, 132].

### 4.3. Re(I) Complex with a Phenanthroline-Dione Ligand

**Figure 8:**
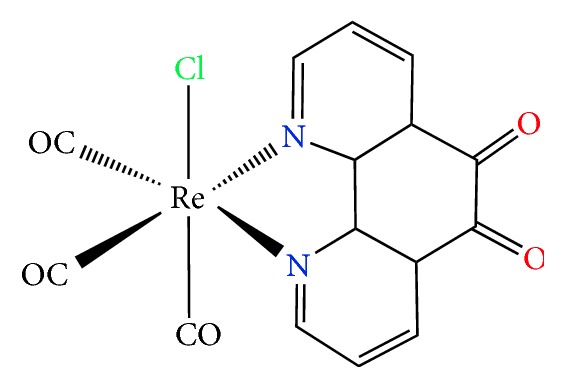
Molecular structure of a Re(I) inhibitor.

Kaplanis and coworkers [142] reported on a Re(I) derivative (Figure 8) of the general type* fac*-[Re(phendione)(CO)_3_Cl] (**Re-1**) (where phendione = 1,10-phenanthroline-5,6-dione) that has been evaluated as inhibitor of PAF-induced aggregation of washed rabbit platelets (IC_50_ = 0.86 *μ*M).

It is to be noted that the starting material [Re(CO)_5_Cl] displayed a better biological activity (IC_50_ = 0.17 *μ*M) as compared to the free ligand phendione (IC_50_ = 0.93 *μ*M) and the Re(I) complex mentioned above. This is in contrast to previous data where coordination of a ligand to a metal ion enhances the inhibitory activity towards PAF aggregation of the metal complex [132, 141]. It may be that** Re-1** complex does not fit into the binding site of PAFR; presumably it dissociates at the extracellular domain and finally the observed activity could be attributed to the activity of the phendione only. In fact,** Re-1** and the free ligand exhibit the same inhibitory effect, while [Re(CO)_5_Cl] does not seem to contribute to the biological activity of the synthesized complex** Re-1**. In this respect, analogous observations have been reported for two new Rh(III) complexes of the formula* mer*-[Rh(L)Cl_3_(MeOH)], where L stands for substituted pyridylquinoline ligands [143]. Coordination of the pyridylquinoline bidentate ligands to the Rh(III) source (RhCl_3_ × 3H_2_O, IC_50_ = 67 *μ*M) improves slightly the biological profile of the metal-based inhibitors, although the Rh(III) metal precursor is not that potent inhibitor as the relevant [Re(CO)_5_Cl] analogue. A plausible answer to the questions raised may be derived with the help of molecular docking theoretical calculations. Finally,** Re-1** and [Re(CO)_5_Cl] showed activity against PAF-basic metabolic enzyme activities in rabbit leukocyte homogenates.

### 4.4. Ru(II) Complexes with Bidentate (N^∧^N) and Tridentate (N^∧^N^∧^N) Nitrogen Based Ligands

**Figure 9:**
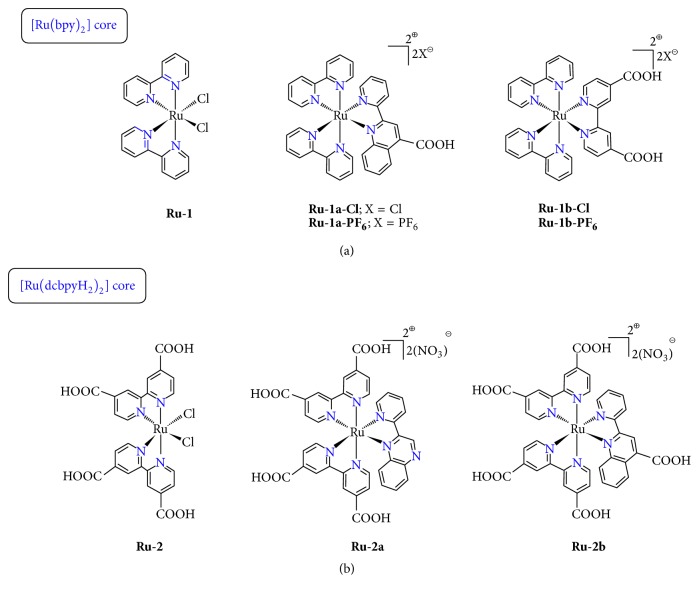
Molecular structures of Ru(II) inhibitors with bidentate ligands.

**Figure 10:**
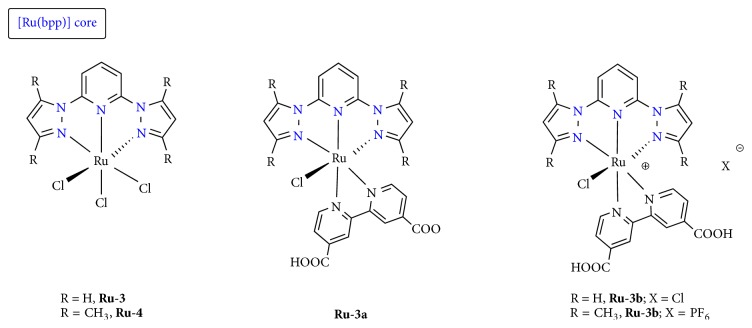
Molecular structures of Ru(III)/(II) inhibitors with tridentate ligands.

Inspired by the previously described results on the field, we wanted to further extend our work to ruthenium(II) coordination compounds and further explore the activity of a variety of coordination compounds containing this metal, as PAF inhibitors [144]. The advantages of ruthenium in a number of biological actions have been well documented [145–147]. Thus a series of octahedral ruthenium(II)/(III) complexes have been tested towards this goal. These substances that have been synthesized by our group incorporate heterocyclic bidentate (N^∧^N) and tridentate ligands (N^∧^N^∧^N) with or without carboxylic acid ancillary functionalities displaying typical octahedral coordination geometries as the majority of ruthenium(II) coordination compounds* (structurally related compounds)*. Among them, those with the -COOH moieties have been mainly tested as sensitizers in third-generation photovoltaic solar cells. Depending on the ruthenium(II) source, these substances can be divided into three categories. The first two comprise the* cis*-[Ru(bpy)_2_(L)]^2+^ [148] (Figure 9(a)) and* cis*-[Ru(dcbpyH_2_)_2_(L)]^2+^ [149] units (where bpy = 2,2′-bipyridine; L = 2-(2′-pyridyl)quinoxaline (L^1^), 4-carboxy-2-(2′-pyridyl)quinoline (L^2^), 2,2′-dipyridine-4,4′-dicarboxylic acid (dcbpyH_2_) (Figure 9(b))), while the third one displays the [Ru(bpp)]^2+^ (bpp is the tridentate ligand 2,6-Bis(1-pyrazolyl)pyridine) [150] and [Ru(bdmpp)]^2+^ (bdmpp = 2,6-bis(3,5-dimethyl-1-pyrazolyl)pyridine) core [144] (Figure 10).

In total, the formulas of the ruthenium complexes examined (studied) are as follows:* cis*-[Ru(bpy)_2_(L)]X_2_ (**Ru**-**1a-Cl**;** Ru**-**1a-PF**_**6**_, L = L^2^ = 4-carboxy-2-(2′-pyridyl)quinoline;** Ru-1b-Cl**,** Ru-1b-PF**_**6**_, L = dcbpyH_2_ = 2,2′-Bipyridine-4,4′-dicarboxylic acid),* cis*-[Ru(dcbpyH_2_)_2_(L)]X_2_ (**Ru-2a**, X = NO_3_, L = L^1^ = 2-(2′-pyridyl)quinoxaline;** Ru-2b**, X = NO_3_, L = L^2^), [Ru(bpp)Cl(dcbpyH)] (**Ru**-**3a**), [Ru(bpp)Cl(dcbpyH_2_)]Cl (**Ru**-**3b**) (bpp = 2,6-Bis(1-pyrazolyl)pyridine), and [Ru(bdmpp)Cl(dcbpyH_2_)]PF_6_ (**Ru**-**4a**) (bdmpp = 2,6-bis(3,5-dimethyl-1-pyrazolyl)pyridine), where* cis*-[Ru(bpy)_2_Cl_2_] (**Ru**-**1**),* cis*-[Ru(dcbpyH_2_)_2_Cl_2_] (**Ru-2**), [Ru(bpp)Cl_3_] (**Ru**-**3**) and [Ru(bdmpp)Cl_3_] (**Ru**-**4**). Results of the biological experiment show that most of the ruthenium complexes are potent inhibitors of PAF with IC_50_ values ranging from micromolar to submicromolar concentrations, extending therefore the range of their applications (Table 4). Inhibition is taking place in a dose-dependent manner. In addition, it has been demonstrated that coordination of the organic ligands to the ruthenium metal center enhances significantly the inhibitory potency against the PAF-induced aggregation. This remark corroborates the results of our previous work based on rhodium(I) and rhodium(III) inhibitors of PAF [124, 132].

Next, we studied the possible effect of the counterion upon ligand exchange reactions. Remarkably, we noticed that, upon substitution reaction of the chloride counteranion of the bpy derivatives** Ru**-**1a-Cl **and** Ru**-**1b-Cl **into the hexafluorophosphate salts,** Ru**-**1a-PF6** (0.48 *μ*M), and** Ru**-**1b-PF6** (0.5 *μ*M), a twentyfold and fourfold increase, respectively, of the PAF inhibitory effect is observed, rendering them more potent against PAF inhibition. This can be attributed in part to the higher inhibitory effect towards PAF of the trifluoroacetyl-analogues, fluoride containing substances, compared to the trichloroacetyl ones [151].

In the second class of complexes, we observed that the inhibitory activity of the ionic compounds** Ru-2a**,** Ru-2b** (IC_50_ values of approximately 0.2 *μ*M) is considerably higher than that of the neutral precursor** Ru-2** (4.5 *μ*M), while an almost 3-fold increase over the bpy derivatives** Ru-1a**-**PF**_**6**_ and** Ru-1b-PF**_**6**_ (0.5 *μ*M) is shown. We can conclude therefore that the presence of the* cis*-[Ru(dcbpyH_2_)_2_]^2+^ core containing -COOH acid groups as auxiliary substituents is more favourable over the classical* cis*-[Ru(bpy)_2_]^2+^ core. The inhibitory effect of the most potent ruthenium(II) compounds reported previously is comparable to that of the well-established rhodium(III) inhibitors,** Rh-1**,** Rh-2, **and** Rh-3 **(0.12 *μ*M, 0.51 *μ*M, and 0.35 *μ*M, resp.) displaying octahedral coordination geometries as well. Presumably this could be attributed to the influence of the electronic configurations and the size of Ru(II) and Rh(III) ions. Remarkably,** Ru-2a** and** Ru-2b** compounds display comparable biological activity (IC_50_ values of 0.18 and 0.24 *μ*M) with rupatadine fumarate, a potent PAF receptor antagonist (0.26 *μ*M) that is used currently (Rupafin) in clinical practice [152]. Within this series, it seems that the ionic compounds are more potent as compared to the neutral precursors (**Ru-1**,** Ru-2**), a result which correlates well with our previous notes on the rhodium-based inhibitors [124, 132]. However, the neutral ruthenium(III) precursor** Ru-3** (IC_50_ = 2.1 *μ*M) displays a slightly higher potency compared to the internal salt** Ru-3a** (3.1 *μ*M) and a more intense inhibitory effect than that of the salt-like complex** Ru-3b** (11.8 *μ*M). In addition, the neutral ruthenium(III) precursor** Ru-4** (IC_50_ = 2.6 *μ*M) is less potent than the ionic complex** Ru-4b** (IC_50_ = 6.4 *μ*M). Moreover,** Ru-3 **and** Ru-4** are more active than the ruthenium(II) compounds Ru-1 (7 *μ*M) and** Ru-2** (4.5 *μ*M), respectively. Obviously, these discrepancies indicate that the inhibitory effect of Ru(III) based inhibitors against PAF is an issue that merits further investigation.

### 4.5. Synopsis of the Metal-Based Inhibitors Reported in This Work

According to the experimental findings, it seems that the nature of the metal center and the nature of the organic ligand attached to it alter substantially the biological action expressed (vide infra). Although the biological probe studied is a complicated system, following simple coordination chemistry principles, we have managed to get some quite helpful structure-activity relationships as mentioned in the conclusions part that follows.

**Figure 11:**
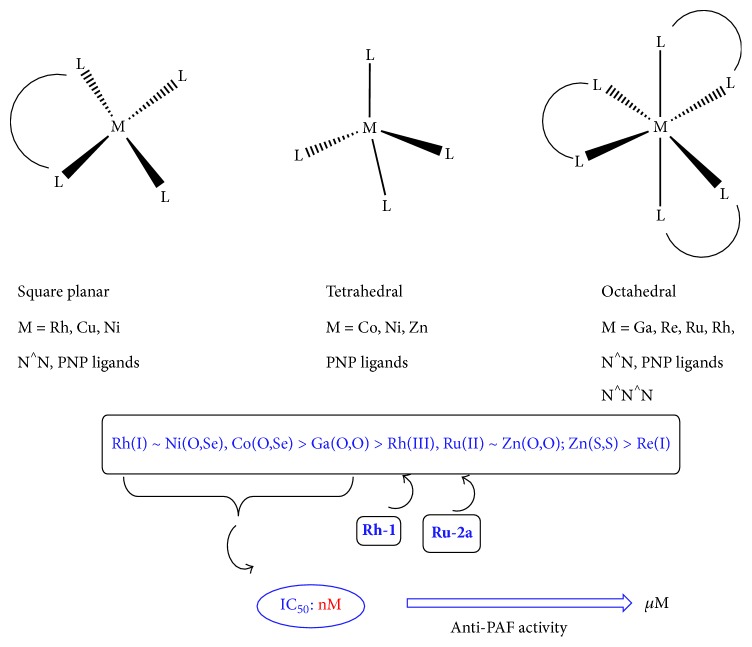
The most potent inhibitors of PAF described in this work.

In general, the square planar Rh(I) complexes incorporating a 2-(2′-pyridyl)quinoxaline ligand (**Rh-1**,** Rh-2**; IC_50_ = 15-16 nM), the pseudotetrahedral Co(II) and Ni(II) complexes (**Co-1**,** Ni-3**, IC_50_ = 18-19 nM), and the octahedral Ga(III) complex bearing chalcogenated imidodiphosphinato ligands (**Ga-1**, IC_50_ = 62 nM) inhibited PAF in the nanomolar scale. Theoretical docking calculations (for the rhodium complexes) are in accord with the experimental findings denoting that** Rh-1**,** Rh-2 **inhibitors could fit in the ligand-binding site of PAF receptor (PAFR).** Rh-1**,** Rh-2**, and** Rh-3 **complexes as well as** Ru-2a**,** Ru-2b **and** Zn-1**,** Zn-2**, and** Re-1** were potent inhibitors in the submicromolar range. A schematic representation of the more potent metal-based inhibitors is given in Figure 11.

## 5. Conclusions

The present minireview describes the latest results on the application of a series of metal complexes with diverse structures and different metal cores as PAF inhibitors in WPRs, a new class of the so-called metal-based inhibitors of PAF. From this work, some preliminary structure-activity relationships have been established based on simplified structural comparison among the library of 30 small molecules investigated. A number of factors such as the counteranion, the total charge of the inhibitor, and the size of the target molecule determine, at least in part, the extent of their inhibitory action. Interestingly, the results show the important role of the metal center and the coordination geometries adopted upon coordination of the organic ligand. Thus, for the rhodium complexes, a straightforward relationship has been addressed, denoting that the square planar organometallic Rh(I) complexes display an inhibitory effect at the nanomolar level than the Rh(III) congeners. The effect of the cyclooctadiene moiety has been analyzed on the basis of theoretical docking calculations, and presumably the improved anti-PAF action has been attributed to the hydrophobic interactions of the cyclooctadiene within the special domains of the PAFR. In addition, the effect of the total charge has been examined, showing that the positively charged Rh compounds and the carboxylic acid containing Ru(II) analogous ([Ru(dcbpyH_2_)_2_]^2+^ core) seem to be the more potent. It may be that, in vitro, these substances display a better permeability across the negatively charged lipid membranes. However, the trend is not followed for the ruthenium bipyridine class ([Ru(bpy)]^2+^ core). On the other hand, on moving to the relevant PNP bifunctional entities, the biological motif alters. The neutral Ni(II)-(O,Se) and Co(I)-(O,Se) analogues now present a strong anti-PAF activity and possibly this may be an effect of the ligand properties.

Upon a thorough study of the molecular structures proposed, we realized that, in most of the cases (not the example of the Re(I) complex), the obtained metal complexes are characterized by an improved biological activity as compared to both the organic precursor and the metal source (synergetic effect). To obtain reasonable* structure-activity *relationships for the other categories tested, as for the rhodium metal compounds, theoretical docking calculations are required.

From the results of this study, one may conclude that indeed metal-based inorganic compounds are a very promising class of anti-PAF and anti-inflammatory drugs. For the rhodium(III) PAF inhibitor** Rh-1**, the moderate cytotoxicity observed in HEK 293 cell lines corroborates the increased anti-inflammatory action observed. Cisplatin that is also active against inflammation is less potent in HEK 293 cell lines. We suggest that this biological effect is due to the overall structural characteristics of** Rh-1** that shows a combined anti-PAF and anticancer activity.

This is of interest, denoting that current organic inhibitors existing could be potentially replaced with a new class of metal-based inhibitors of PAF in the near future. Finally, the results of this study could help us to create a database set of coordination compounds with potential anti-inflammatory activity. In such a way, we could roughly predict or estimate the potency of other new metal-based substances against PAF, displaying similar molecular structures. Thus the preliminary results reported could provide us with the required knowledge so as to rationally design more potent anti-inflammatory drugs in the future.

## Supplementary Material

Chemical structures of PAF antagonists isolated from wines and oils.

## Figures and Tables

**Table 1:** IC_50_ (*μ*M, final concentration) of various PAF inhibitors that induces 50% inhibition against PAF aggregation in washed rabbit platelets (WRPs). IC_50_ (*μ*L) values are expressed in microliters of initial volume of oil that induces 50% inhibition against 1 × 10^−11^ M PAF final concentration in the aggregometer cuvette.

PAF inhibitor	IC_50_	Reference
Ginkgolides		
BN 52021	3.6	[13]
BN 52020	9.7	[13]
BN 52022	38	[13]
Alpha-bulnesene	0.024	[17]
Cedrol	13	[29]
Paricalcitol	0.3	[100]
Di-hydroxy-chimyl-alcohol (pine pollen)	4.5	[27]
Olive oil	6.5 *μ*L	[74]
Sunflower	21 *μ*L	[88]
Sesame oil	150 *μ*L	[88]

**Table 2:** IC_50_ data (*μ*M) final concentration in the aggregometer cuvette for rhodium PAF inhibitors towards WRPs.

Number	Rhodium complexes	IC_50_
**Rh-1**	*cis*-[Rh(L)_2_Cl_2_]Cl	0.12 ± 0.11
**Rh-2**	*cis*-[Rh(L1)_2_Cl_2_]Cl	0.51 ± 0.23
**Rh-3**	*cis*-[Rh(L2)_2_Cl_2_]Cl	0.35 ± 0.20
**Rh-4**	*mer*-[Rh(L)Cl_3_(MeOH)]	2.6 ± 2.0
**Rh-5**	[Rh(L)(cod)]Cl	0.016 ± 0.015
**Rh-6**	[Rh(L)(cod)]NO_3_	0.015 ± 0.015
—	cisplatin	0.55 ± 0.22

**Table 3:** IC_50_ data (*μ*M) final concentration in the aggregometer cuvette for metal complexes bearing PNP ligands as PAF inhibitors.

Number	PNP complexes	ΙC_50_
**Cu-1**	Cu{(OPPh_2_)(OPPh_2_)N-O,O}_2_	~1.0
**Zn-1**	Zn{(OPPh_2_)(OPPh_2_)N-O,O}_2_	0.54
**Zn-2**	Zn{(SPPh_2_)(SPPh_2_)N-S,S}_2_	0.36
**Zn-3**	*Ζ*n{(OPPh_2_)(SePPh_2_)N-O,Se}_2_	1.11 ± 0.22
**Ga-1**	Ga{(OPPh_2_)(OPPh_2_)N-O,O}_3_	0.062 ± 0.045
**Ni-1**	Ni{(Ph_2_P)_2_N-S-CHMePh-P,P}Cl_2_	~16
**Ni-2**	Ni{(Ph_2_P)_2_N-S-CHMePh-P,P}Βr_2_	~3.0
**Ni-3**	Ni{(OPPh_2_)(SePPh_2_)N-O,Se}_2_	0.019 ± 0.006
**Co-1**	Co{(OPPh_2_)(SePPh_2_)N-O,Se}_2_	0.018 ± 0.005

**Table 4:** IC_50_ data (*μ*M) of various Ru(II)/(III) PAF inhibitors.

Number	Complexes	IC_50_
**Ru-1**	[Ru(bpy)_2_Cl_2_]	7.0 ± 0.7
**Ru-1a-Cl**	[Ru(bpy)_2_(L^2^)]Cl_2_	11 ± 1
**Ru-1a-PF**_**6**_	[Ru(bpy)_2_(L^2^)](PF_6_)_2_	0.48 ± 0.06
**Ru-1b-Cl**	[Ru(bpy)_2_(dcbpyH_2_)]Cl_2_	1.2 ± 0.1
**Ru-1b-PF**_**6**_	[Ru(bpy)_2_(dcbpyH_2_)](PF_6_)_2_	0.50 ± 0.05
**Ru-2**	[Ru(dcbpyH_2_)_2_Cl_2_]	4.5 ± 0.5
**Ru-2a**	[Ru(dcbpyH_2_)_2_(L^1^)](NO_3_)_2_	0.18 ± 0.01
**Ru-2b**	[Ru(dcbpyH_2_)_2_(L^2^)](NO_3_)_2_	0.24 ± 0.03
**Ru-3**	[Ru(bpp)Cl_3_]	2.1 ± 0.2
**Ru-3a**	[Ru(bpp)(dcbpyH)Cl]	3.1 ± 0.3
**Ru-3b**	[Ru(bpp)(dcbpyH_2_)Cl]Cl	11.8 ± 0.1
**Ru-4**	[Ru(bdmpp)Cl_3_]	2.6 ± 0.3
**Ru-4b**	[Ru(bdmpp)(L^4^)Cl](PF_6_)	6.4 ± 1.1
