# Supplementary material for: A Review on Platelet Activating Factor Inhibitors: Could a New Class of Potent Metal-Based Anti-Inflammatory Drugs Induce Anticancer Properties?
Source: Bioinorg Chem Appl. 2017 Mar 28;2017:6947034. doi: 10.1155/2017/6947034 (PMC5387815; doi:10.1155/2017/6947034)

**Supplementary data**

**For**

**A mini-review on Platelet Activating Factor (PAF) Inhibitors. Could a new class of potent Metal-based anti-inflammatory drugs induce Anticancer Properties?**

Vasiliki D. Papakonstantinou^a^, Nefeli Lagopati^b^, Effie C.Tsilibary^b^, Constantinos A. Demopoulos^a^, and Athanassios I. Philippopoulos^c^*

*^a^ Laboratory of Biochemistry, Faculty of Chemistry, National and Kapodistrian University of Athens, Athens 15771, Greece*

*^b^ Ιnstitute of Biosciences & Applications, NCSR Demokritos, Aghia Paraskevi,
15310 Greece*

*^c^ Laboratory of Inorganic Chemistry, Department of Chemistry, National and Kapodistrian University of Athens, Panepistimiopolis Zografou 15771, Athens, Greece*

* Corresponding author: Prof. A. I. Philippopoulos. Tel.: +30-210-7274697; Fax: +30-210-7274782; e-mail: [atphilip@chem.uoa.gr](mailto:atphilip@chem.uoa.gr)

**Figure S1**. Structures of platelet-activating factor antagonists isolated from wines. A) white wine (Ambelon, Robola); B) white must (Robola); C) white wine (Ambelon, Robola); D) red wine (Cabernet Sauvignon).


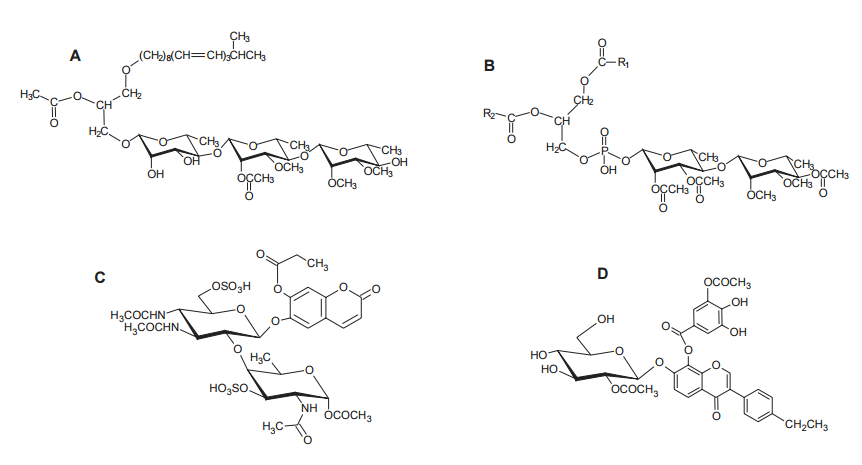


**Figure S2**. Structure and MS fragmentation of platelet-activating factor antagonists isolated from A) corn oil, B) sesame oil and C) sunflower oil , D) olive oil and E) olive pomace.


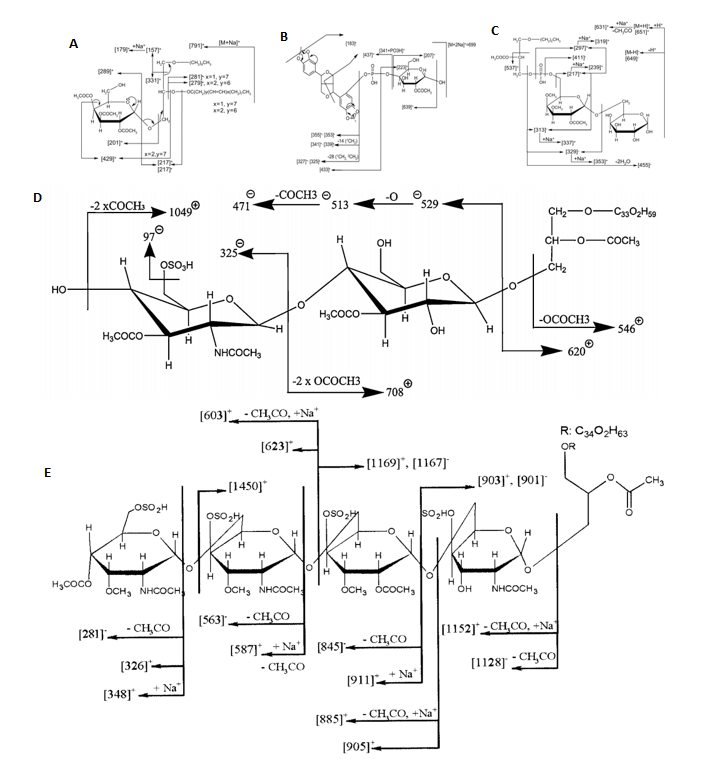

Supplement: Supplementary file 1 — Chemical structures of PAF antagonists isolated from wines and oils. [file 6947034.f1.docx]
